# Initial Validation of the Mindful Presence Scale: The Issue of the Construal Level of Scale Items

**DOI:** 10.3389/fpsyg.2021.626084

**Published:** 2021-07-21

**Authors:** Attila Lengyel, Danica Keczeli, Róbert Orosz, Zoltán Bács, Anetta Müller, Szilvia Szőke, Éva Bácsné Bába

**Affiliations:** ^1^Department of Tourism Management and Catering, Institute of Rural Development Tourism and Sports Management, Faculty of Economics and Business, University of Debrecen, Debrecen, Hungary; ^2^Department of Sport Economics and Management, Institute of Rural Development, Tourism and Sports Management, Faculty of Economics and Business, University of Debrecen, Debrecen, Hungary; ^3^Institute of Psychology, Faculty of Humanities, University of Debrecen, Debrecen, Hungary; ^4^Institute of Accounting and Finance, Faculty of Economics and Business, University of Debrecen, Debrecen, Hungary; ^5^Department of Research Methodology and Statistics, Institute of Sectorial Economics and Methodology, Faculty of Economics and Business, University of Debrecen, Debrecen, Hungary

**Keywords:** methodological issues, construal level, scale validation, short scale, trait mindfulness, mindfulness

## Abstract

Our research has two main aims. It undertakes the validation of a six-item trait mindfulness scale called Mindful Presence Scale (MPS), which measures central aspects of mindfulness. For the first time in mindfulness literature, the construal level of scale items is also examined. Four questionnaire-based online studies were conducted. Study 1 drew three samples (*n*_*n*_ = 391, *n*_*p*_ = 215, and *n*_*b*_ = 235) from the students at the University of Debrecen. It examined the factor structure, reliability, and internal consistency of the three differently worded scale versions. The positively worded scale version (MPSp) yielded a stable two-factor structure and demonstrated the best psychometric properties. Study 2 performed a confirmatory factor analysis on a sample drawn from public employees across the country (*n*_*cfa*_ = 420). The two-factor solution in Study 1 was confirmed. χ^2^ tests were not significant, and fit indices were excellent. There was no significant difference between the high-level construal subscale (F_hlc_) and the low-level construal subscale (F_llc_) in terms of factorial stability. Participant of Study 3 were students who did not take part in Study 1. The sample (*n*_*inv*_ = 250) was tested for measurement invariance across gender. The scaled results supported strong/threshold invariance. Study 4 tested concurrent validity with 10 concurrent instruments. A sample of secondary school teachers (*n*_*con*_ = 128) was tested by examining Spearman's rank order correlations. There was a significant difference in how the F_hlc_ and F_llc_ subscales predicted scores of some of the concurrent instruments. Further research is warranted into how the construal level of mindfulness scale items affects the recollection of the mindful experience. Overall, MPS_p_ proved to be a valid short mindfulness measure.

## Introduction

Mindfulness is a multifaceted construct with many contested definitions that can demonstrate both considerable overlaps and contradictions (Van Dam et al., 2018; Anālayo, 2019). Instead of embracing any of the definitions of the past three decades, the present study attempts to identify a construct which is proposed to constitute the basis for the evolution of all other facets of mindfulness. Based on this core conceptualization of mindfulness, a short self-report instrument is developed as the operationalization of the construct and an initial validation procedure of the scale is carried out. Although numerous self-report measures of trait mindfulness have been developed and validated (Baer et al., 2004, 2008; Walach et al., 2006; Feldman et al., 2007; Cardaciotto et al., 2008; Chadwick et al., 2008; Davis et al., 2009; Bergomi et al., 2013; Frank et al., 2016; Pratscher et al., 2019) since the Mindful Attention and Awareness Scale (hereafter MAAS) was first tested (Brown and Ryan, 2003), apart from the five-item MAAS (Van Dam et al., 2010; Osman et al., 2016; Smith et al., 2017) and six-item MAAS (Black et al., 2012) no other really short (under 10 items) trait mindfulness scale has been validated before. As a second goal, our study attempts to investigate whether the construal level of the scale items has any significant effect on the recollection of mindful episodes by respondents of the self-report questionnaire.

Mindfulness is a metacognition-based dynamic process involving the ability to intentionally regulate and control attention (Wells, 2000, 2002; Teasdale et al., 2002; Sugiura, 2004; Norman, 2017; Bernstein et al., 2019). This dynamism also entails, with the exclusion of very experienced meditators, a constant fluctuation between mindful and mindless states. Based on this fact, the question arises: How does mindfulness begin? According to Bishop et al., “Mindfulness begins by bringing awareness to current experience…” (Bishop et al., 2004, p. 232). In Bishop et al.'s (2004) operational definition, mindfulness is a two-component hierarchical process where the first step is a samatha-type attention control employed to bring back and anchor attention in the present moment. The special importance of being able to bring attention back to the present moment and anchor it there has been supported by theoretical arguments and evidenced by numerous empirical studies (Baer et al., 2008; Dreyfus, 2011; Gethin, 2011; Christopher et al., 2012; Lilja et al., 2013; Siegling and Petrides, 2016; Watson-Singleton et al., 2018). Brown and Ryan states, “This present-centered attention–awareness is, in our view, foundational to mindfulness…” (Brown and Ryan, 2003, p. 824). Cardaciotto et al. (2008) consider awareness a “central component” of mindfulness (Cardaciotto et al., 2008, p. 220). Based on what has been said so far, we propose that *scale items that measure the ability to bring attention to and anchor it in the present moment and direct it intentionally to anything that arises in the space of consciousness, should be categorized under the term Mindful Presence (MP)*. Once MP is established and maintained, it can serve as a springboard to the second step in the mindfulness meditation process, which involves the open, curious, non-reacting, non-judgmental witnessing of anything that is arising and unfolding (vipassana-type meditation) in the space of consciousness, that is, experiencing non-identification with the sensual information, thoughts, feelings, images, or desires. Without stabilizing attention and establishing an MP, no insight is possible. You cannot let things go without even becoming aware that you are holding on to them. Bishop et al. (2004) emphasize that “skills” such as being able to let things arise (openness, curiosity, and non-judgment) and letting them pass (non-reaction) as well as the ability to exercise virtues such as compassion, wisdom, or moderation, to mention a few considered necessary in Buddhist practice, are “correlates” or “benefits” of the MP established by anchoring attention in the present moment.

Trait mindfulness instruments developed so far aim to tap into various dimensions or facets of mindfulness, including observing, non-judging, non-reacting, acceptance, acting with awareness, describing, attention, present focus, and awareness. Dimensions such as observing, acting with awareness, attention, present focus, and awareness overlap with MP. Instead of using various terms for these overlapping facets, *we suggest that their items are better categorized according to their level of abstraction or construal level* (Liberman and Trope, 1998; Trope and Liberman, 2010). All the items in Table 1, whether worded in a general (abstract) or a concrete (action-specific) way, tap into the construct of MP.

**Table 1:** Relevant sample items from scales used in the validation process.

**Scale and subscale**	**High-level construal items**
CAMS-R Attention subscale: Item 11.	I am able to focus on the present moment.
FFMQ Acting with Awareness subscale: Item 38	I find myself doing things without paying attention.
MAAS: Item 3	I find it difficult to stay focused on what's happening in the present.
**Scale and subscale**	**Low-level construal items**
FFMQ Observe subscale: Item 1	When I'm walking, I deliberately notice the sensations of my body moving.
PHLMS Awareness subscale: Item 5.	When I shower, I am aware of how the water is running over my body.
FMI Mindful Presence subscale: Item 3.	I sense my body, whether eating, cooking, cleaning or talking.

While all items in Table 1 can be considered operationalizations of MP, they represent different levels of construal. For example, PHLMS Awareness subscale item 5 is considered to be low-level construal since it describes the concrete daily activity of having a shower. In comparison, CAMS-R Attention subscale item 11 is a statement about a general ability; thus, it is categorized as a high-level construal item. It has to be noted that construal level is a continuum from the local (specific, concrete) to the global (abstract, general) with no clear-cut boundaries (Alter and Oppenheimer, 2008). Hence, the terms “low-level construal” and “high-level construal” in the present paper are relative terms and in fact denote higher and lower levels of construal.

**Figure 1:**
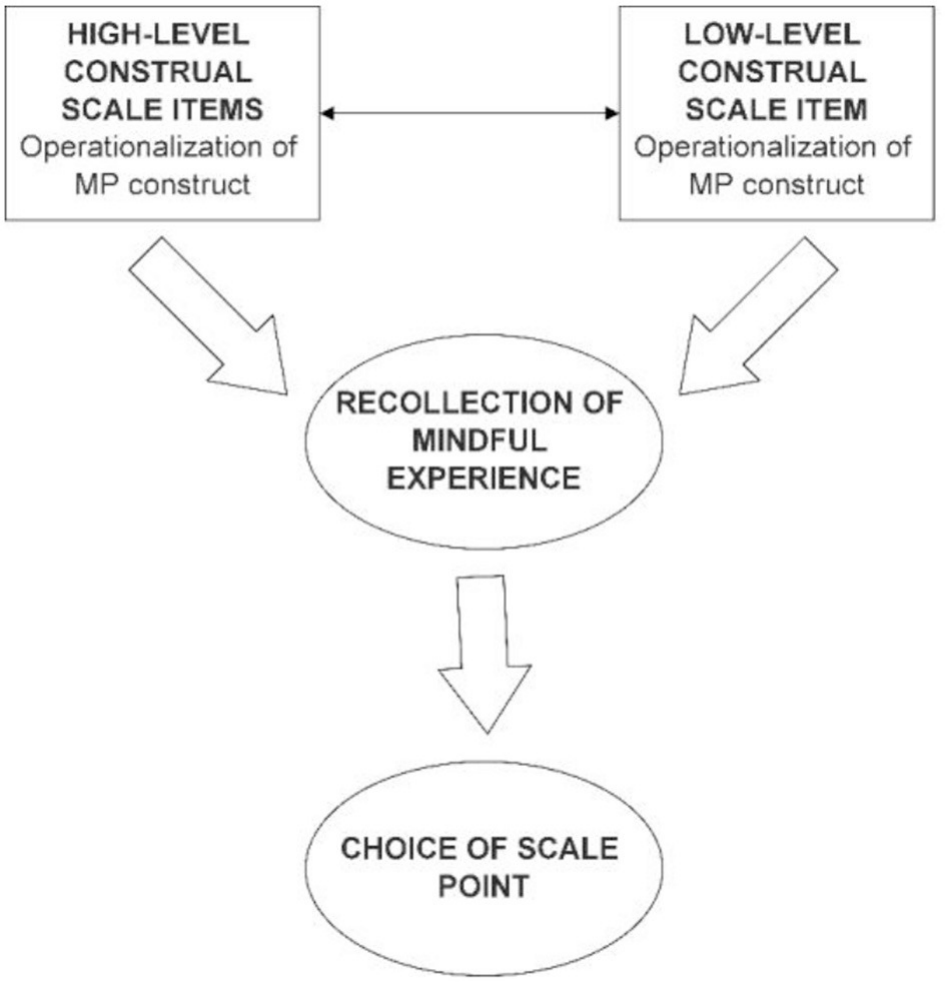
Hypothesized effect of construal level on the recollection of mindful experience.

The level of construal might affect psychological constructs (Vess et al., 2011; Gong and Medin, 2012; Mantzios and Wilson, 2014), and mindfulness may also have an effect on the level of construal (Heeren et al., 2009; Chan and Wang, 2019). The question of how the construal level of self-report items might affect the recollection of mindful moments by respondents is practically unresearched to date. High-level and low-level construal items of the MP construct might influence how mindful behavior is recollected by respondents. The conceptual framework of investigating the possible effects of construal level of scale items on recollection of mindful episodes is shown in Figure 1.

We hypothesized that the high- and low-level construal items would perform differently in the statistical procedures. We also expected that because both high- and low-level construal items are operationalizations of MP as a single construct, there would be signs of unidimensionality.

## Materials and Methods

### Item Generation and Development of the Measuring Instrument

This initial phase consisted of four steps:

1. *Item generation*. Deductive approach was used to create an initial pool of 58 MP-related items from existing mindfulness scales and MP-related items created by the authors based on the operational definition of MP given in the Introduction. All items were categorized into either the high-level or the low-level construal group depending on whether they described a specific action (e.g., having a shower) or a general skill (e.g., ability to focus on what one is doing).
2. *Development of the measurement instrument*. Content validity of scale items was determined by an expert panel of five judges, two psychiatrists using mindfulness-based therapies in their clinical practice, and three mindfulness meditation experts. The two psychiatrists are certified mindfulness trainers who have been running Mindfulness Based Cognitive Therapy (MBCT) groups for over 5 years. They both graduated from a mindfulness trainer course certified by the Oxford Mindfulness Center. Two of the meditation experts are senior leaders of Buddhist meditation communities with decades of meditation experience in both vipassana- and samatha-type techniques. The third expert is a doctor and certified Mindfulness Based Stress Reduction (MBSR) trainer running courses mainly for people struggling with addiction problems.
  Similar to the procedure used by Cardaciotto et al. (2008), judges rated high-level and low-level construal items separately on a 5-point Likert-type rating scale (1= very poor, 2 = poor, 3 = fair, 4 = good, and 5 = very good) based on how well, in their opinion, the item measured MP. Interclass correlation (ICC) coefficient was calculated using SPSS version 25. A high degree of interrater agreement was found: average measure = 0.842, lower bound = 0,801, upper bound = 0.877 with a 95% confidence interval [*F*_(199,398)_ = 6.349, *p* < 0.001]. Based on these results, three items from both pools with the highest mean ratings were chosen to form the basis of the Mindful Presence Scale (MPS). Five of the six items in MPS are differently worded variations of the already-existing mindfulness scale items. Although all items are operationalizations of the theoretical construct of MP described in the Introduction, we hypothesized that the construal level of the items is an aspect that will manifest in the factor analytical procedure. The scale consists of six items because we hypothesized that the high- and low-level construal scale items would gravitate toward two hidden variables (factors) and the minimum acceptable number of indicators per factor for relatively small samples with adequately high factor loadings is three (Wolf et al., 2013; Koran, 2020).
3. *Handling open methodological issues*. Some open methodological issues were dealt with in this phase. Ambiguity of scale items may seriously affect the validity (Carmody et al., 2009; Park et al., 2013). Many of the scale items of the frequently used mindfulness scales are abstract without any context. As an example, MAAS item 11 is a general statement “I find myself listening to someone with one ear, doing something else at the same time.” We can interpret this statement in two typical everyday contexts, leisure, and work. The distinction seems rather important as the level of agreement or disagreement with the item might change radically. The questionnaire used in this study avoids this pitfall by giving clear instructions to the respondents to think about the scale items imagining that: *they are at home, alone, spending their free time*.
  The other open issue to be resolved was the question of positively and negatively worded items. There are authors who argue for negatively worded items (Brown and Ryan, 2003), or positively worded items (Hartley, 2014), or some mixture of them (Baer et al., 2008). The presently used mindfulness scales show considerable differences in their approach to item wording. The authors decided on a strategy to initially screen all the three above-discussed wording approaches in connection with the MPS items using factor analytic and reliability checking procedures and move on to the validation process with the scale version that has shown the best psychometric properties. Similar to MAAS, a six-point rating scale was used in the questionnaire with scale points ranging from 1 (almost never) to 6 (almost always).
4. Pilot test to check *face validity*. A sample of convenience (*n*_1_ = 176) from Facebook was used to test the face validity of the positively and negatively worded scale items. Of the sample, 72.6% had college or university degrees or both. Based on the feedback received from the respondents, the wording of test items was modified and finalized.

### Overview of Validation Procedure

**Figure 2:**
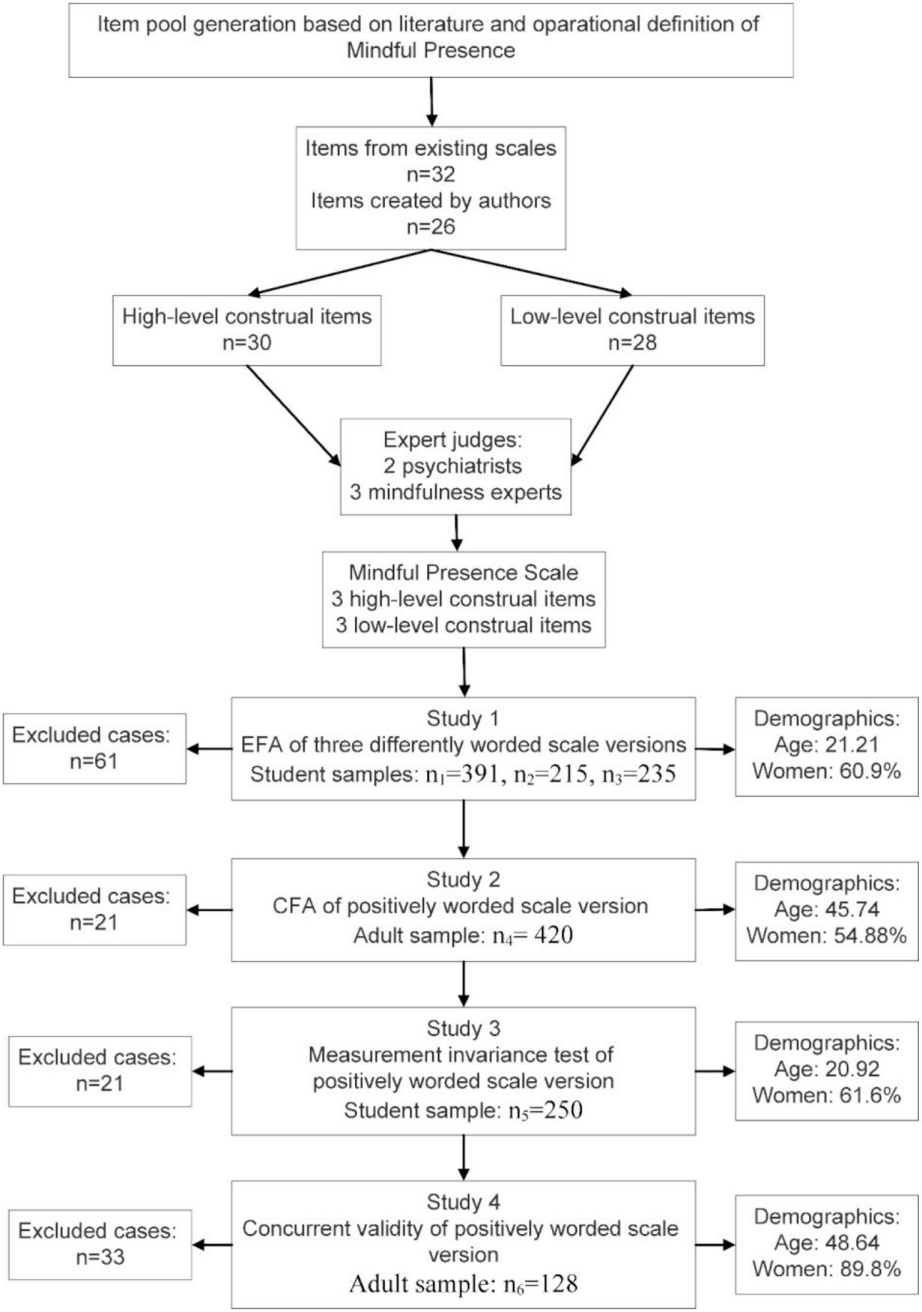
Study flow.

The validation process consists of four studies involving samples of various base populations. In Study 1, samples were drawn from among undergraduate students at the University of Debrecen *via* convenience sampling. Study 1 used three samples (*n*_1_ = 391 negatively worded version, *n*_2_ = 215 positively worded version, and *n*_3_ = 235 balanced version). The three sets of data were tested with exploratory factor analysis (EFA) as well as reliability and internal consistency indices. In Study 2, a sample was drawn from a pool of local government employees across the country (*n*_4_ = 420) to validate the findings of Study 1 through confirmatory factor analysis (CFA). In Study 3, a sample of students who have not taken part in Study 1 (*n*_5_ = 250) studying at the University of Debrecen was tested to examine measurement invariance across gender. In Study 4, the MPS version with the best psychometric properties was included in the validation questionnaire with 10 concurrent instruments. This validation questionnaire was sent out to teachers of secondary schools. The sample (*n*_6_ = 128) served as a basis for testing concurrent validity. Figure 2 shows the study flow.

As EFA and CFA can be viewed as two ends of a continuum in terms of imposing or not imposing restrictions on the solutions, they are rather complimentary than mutually exclusive (Hoyle, 2000); thus, both methods are applied in Study 1 and Study 2, respectively. In order to support construct validity, measurement invariance (special application of CFA) is tested in Study 3. Finally in Study 4, convergent and discriminant validity of the scale is tested using concurrent instruments.

The data were collected *via* self-reported online questionnaires that contained the scale items and questions about demographic information. Before completing the questionnaire, participants were informed about the goal of the study, the anonymity of the questionnaire, and the ethical approval that had been obtained from the Committee of Scientific Ethics at the University of Debrecen (GTKDH/52/2021:15.13.). They were informed that data management was in accordance with European Union directives and Hungarian laws regulating the handling of personal data. Before they were able to proceed to completion, they were asked to give their informed consent that the data they provided could be used for scientific analysis and the results published in scientific journals. For the statistical analysis, both commercial (SPSS version 25) and noncommercial software (R statistical packages and FACTOR software) were used.

#### Study 1: Factor Structure, Reliability, and Internal Consistency of the Three Scale Versions

##### Participants

Undergraduate students studying at the University of Debrecen were recruited to complete the online self-reported questionnaires containing the scales MPS_p_, MPS_n_, and MPS_b_. For each scale version, 1,000 e-mails containing the link to the questionnaire and the brief introduction of the research were sent out. Out of the 3,000 questionnaires, 902 were sent back. Forty-two were excluded from the analysis as they were either empty or missing too much information. Sixteen respondents marked the “I do not approve” option for the future scientific use of the data and three questionnaires were judged to be inadequate as the respondents marked the same scale point all through the questionnaire. As the samples were drawn from the same base population, they (*n*_1_ = 391, *n*_2_ = 215, *n*_3_ = 235) showed very similar demographic characteristics concerning age, gender, and training program (2-year vocational, bachelor, masters). In n_1_, the mean age for respondents was 21.74 years (*SD* = 10.84), and the proportion of women was 54.80%. In n_1_, the three training programs were represented as follows: 2-year vocational = 34.3%, bachelor = 41.7%, and masters = 24%. In n_2_, the mean age for respondents was 20.81 years (*SD* = 4.85), and the proportion of women was 60.10%. In n_2_, the three training programs were represented as follows: 2-year vocational = 27.3%, bachelor = 49.2%, and masters = 23.5%. In n_3_, the mean age for respondents was 21.08 years (*SD* = 6.61), and the proportion of women was 67.8%. In n_3_, the three training programs were represented as follows: 2-year vocational = 37.9%, bachelor = 38.2%, and masters = 21.9%. The sample sizes were all above the typically recommended minimum of *n* = 200 in the EFA literature (Fabrigar and Wegener, 2011; Jung and Lee, 2011), and the subject-to-variable ratios were well above the conservative recommendation of 20:1 (Osborne et al., 2014) even in the smallest (n_2_) sample (36:1).

##### Data Analysis

For factor extraction, robust diagonally weighted least squares (RDWLS) (Mîndrila, 2010) was employed as there are only three indicators per factor, and a relatively small sample size (Forero et al., 2009). Dimensionality tests were run using FACTOR software, random.polychor.pa R package (Jordan et al., 2019), nFactors R package (Raiche, 2010), psych R package (Revelle, 2016), and RgenData R package (Ruscio and Roche, 2012). As the factors were expected to be correlated, oblique rotation method (direct oblimin) was employed (Verhaeghen and Aikman, 2020). For the minimum percentage of variance accounted for by latent variables, a 60% cutoff value was used (Hair et al., 2009). Special EFA indices were computed to provide further information about various aspects of the EFA solutions (Bentler, 1977; Lorenzo-Seva, 2003; Finch et al., 2008; Rodriguez et al., 2016; Bado et al., 2018; Ferrando and Lorenzo-Seva, 2018).

Alpha's excessive use in psychometric literature has been criticized by many studies (Morera and Stokes, 2016; McNeish, 2018). There are several alternatives to alpha recommended by the literature such as GLB and Omega (Sijtsma, 2009; Watkins, 2017), ordinal alpha (Basto and Pereira, 2012; Morera and Stokes, 2016), or coefficient H (McNeish, 2018). The authors adopted an approach of reporting all above-mentioned indicators of consistency and reliability.

##### Results and Discussion

As the data proved to be non-normal by both the Kolmogorov–Smirnov and the Shapiro–Wilk tests (*p* > 0.001), the polychoric correlation matrices were computed for the three scale versions in FACTOR software using Bayesian estimation. Factorability tests based on the polychoric matrices resulted: MPSp (determinant of the matrix = 0.09; Bartlett's statistic = 499.90, *df* =15; *p* = 0.00; Kaiser–Meyer–Olkin test = 0.74), MPSn (determinant of the matrix = 0.49; Bartlett's statistic = 272.80, *df* = 15; *p* = 0.00; Kaiser–Meyer–Olkin test = 0.72), MPSb (determinant of the matrix = 0.01; Bartlett's statistic = 1,081.70, *df* = 66; *p* = 0.00; Kaiser–Meyer–Olkin test = 0.79). For comparison, most tests were run on both the Pearson and the polychoric matrices. For MPSp and MPSn, there is a strong agreement between the employed dimension testing methods suggesting two dimensions. For MPSb, most methods suggested four dimensions. In the subsequent EFA, only these factor models were tested. Factor analysis was done in FACTOR software. Table 2 shows the pattern matrices of the oblique solution for the three scale versions with factor loadings and cross-loadings ≥0.30.

**Table 2:** Result of EFA.

**Variables**	**MSPp loadings**		**Com**.	**MPSn loadings**		**Com**.	**MPSb loadings**				**Com**.
	${\text{F}}_{\text{hlc}}^{\text{a}}$	${\text{F}}_{\text{llc}}^{\text{b}}$		**-F_**hlc**_**	**-F_**llc**_**		**F_**hlc**_**	**F_**llc**_**	**-F_**hlc**_**	**-F_**llc**_**	
hlc1	0.75	–	0.62	–	–		0.67	–	–	–	0.66
hlc2	0.79	–	0.63	–	–		0.77	–	–	–	0.69
hlc3	0.90	–	0.76	–	–		0.71	–	–	–	0.62
llc1	–	0.80	0.62	–	–		–	0.81	–	–	0.75
llc2	–	0.69	0.48	–	–		–	0.77	–	–	0.77
llc3	–	0.63	0.46	–	–		–	0.79	–	–	0.67
-hlc1	–	–		0.50	–	0.37	–		0.36	0.36	0.40
-hlc2	–	–		0.67	–	0.39	–	–	0.79	–	0.63
-hlc3	–	–		0.46	–	0.32			0.56	–	0.52
-llc1	–	–		–	0.53	0.33	–	–	–	0.62	0.50
-llc2	–	–		–	0.65	0.41	–	–	–	0.60	0.63
-llc3	–	–		–	0.56	0.32	–	–	–	0.81	0.66

*^a^F_hlc_, factor of high-level construal variables; ^b^F_llc_, factor of low-level construal variables. Com., communalities. Loadings lower than 0.30 are omitted*.

F_hlc_ denotes the factor with high-level construal scale items loading onto it, and F_llc_ denotes the factor with low-level construal scale items loading onto it. What is immediately apparent from Table 2 is that all three scale versions, with the exception of the first high-level construal scale item of the negatively worded subscale of MPSb, demonstrate a clear factor structure. In the case of MPSp and the MPSp part of MPSb, both the high-level and low-level construal variables load strongly on the two factors. The MPSn and the MPSn part of MPSb yielded weaker factor loadings. Table 3 contains indices that provide further information about various aspects of the EFA solutions. At first glance, it seems obvious that the MPSp two-factor solution and the MPSp part of MPSb come closest to the cutoff criteria, while MPSn and the MPSn part of MPSb do rather poorly.

**Table 3:** Indices to assess the quality of EFA factor solutions.

**Indices**	**Cutoff values**	**MSPp**		**MPSn**		**MPSb**			
		${\text{F}}_{\text{hlc}}^{\text{i}}$	${\text{F}}_{\text{llc}}^{\text{j}}$	**-F_**hlc**_**	**-F_**llc**_**	**1F_**hlc**_**	**3F_**llc**_**	**4-F_**hlc**_**	**2-F_**llc**_**
Interfactor correlations		0.46		0.44		0.29^k^			
Explained variance	≥60%	0.72		0.57		0.74			
FDI^a^	≥0.9	0.93	0.88	0.79	0.81	0.92	0.86	0.94	0.89
GH^b^-latent	≥0.8	0.87	0.78	0.62	0.66	0.85	0.75	0.89	0.79
GH-observed	≥0.8	0.85	0.76	0.58	0.63	0.85	0.79	0.88	0.67
SR^c^	≥2	2.60	1.90	1.29	1.39	2.36	1.72	2.86	1.97
UniCo^d^	≥0.95	0.91		0.92		0.77			
ECV^e^	≥0.85	0.73		0.74		0.67			
MIREAL^f^	≤ 0.3	0.39		0.28		0.39			
S^g^	≥0.8	0.99		0.99		0.98			
LS^h^	≥0.8	0.85		0.55		0.50			

a *FDI, Factor Determinacy Index*;

b *GH, Generalized Construct Replicability Index*;

c *SR, Sensitivity Ratio*;

d *UniCo, Unidimensional Congruence*;

e *ECV, Explained Common Variance*;

f *MIREAL, Mean of Item REsidual Absolute Loadings*;

g *S, Bentler's simplicity index*;

h *LS, Loading simplicity index*;

i *F_hlc_, factor of high-level construal variables*;

j *F_llc_, factor of low-level construal variables*;

k *Average value, interfactor correlations ranged 0.14–0.6*.

It must be noted that, in spite of the two-factor structure, based on the indices measuring the scale's closeness to unidimensionality (UniCo, ECV, MIREAL), MPSp seems to demonstrate that there is a common underlying factor. Its unidimensional character is supported by fact that the first eigenvalue accounts for 53.15% of the 72.47% total explained variance. Factor scores of the two MPSp factors are strongly correlated with the MPSp composite scores (*F*_*hlc*_ *r* = 0.81, *p* < 0.05, *F*_*llc*_ *r* = 0.83, *p* < 0.05) and the interfactor correlation is of medium strength (*r* = 0.46, *p* < 0.05). It has to be noted that in the subsequent studies, the interfactor correlation between the high-level and low-level construal factors ranged from 0.43 to 0.62, *p* < 0.05.

Although there seems to be a difference between the high-level construal (F_hlc_) and the low-level construal (F_llc_) variables according to data in Table 3, in the other three samples examined in subsequent studies (n_4_, n_5_, n_6_), there was no significant difference in EFA indices of the two subscales.

Table 4 shows the scale reliability and internal consistency values. In the case of a multidimensional model sum scores for the total scale are not computed only subscale scores.

**Table 4:** Reliabilities of the three scale versions.

**Indices**	**MSPp**		**MPSn**		**MPSb**			
	**F_**hlc**_**	**F_**llc**_**	**-F_**hlc**_**	**-F_**llc**_**	**F_**hlc**_**	**F_**llc**_**	**-F_**hlc**_**	**-F_**llc**_**
α	0.82	0.71	0.56	0.57	0.79	0.85	0.63	0.72
o^a^.α	0.78	0.71	0.57	0.60	0.80	0.86	0.65	0.72
Revelle's Ω	0.82	0.72	0.57	0.58	0.80	0.85	0.66	0.72
o.Ω h^b^	0.78	0.71	0.57	0.60	0.79	0.86	0.65	0.72
glb	0.83	0.73	0.57	0.59	0.80	0.85	0.67	0.72
H	0.83	0.73	0.57	0.59	0.81	0.85	0.69	0.72

a *o., ordinal, the other estimates assume interval level*;

b *Ω h, omega hierarchical*.

Scale reliability values depicted in Table 4 seem to follow a consistent pattern. In the case of MPS_p_, all reliability indices are higher for the high-level construal subscale (F_hlc_). However, in MPS_n_ and MPS_b_, the exact opposite holds true as values for the low-level construal subscale (F_llc_) are consistently higher than values for the high-level construal subscale (F_hlc_). In subsequent studies, although to a lesser extent, the same pattern emerged. In n_6_ and n_4_, however, in n_5_ it was the low-level construal subscale that demonstrated significantly better reliability values.

Based on the EFA loading values, EFA indices, and the reliability and consistency values, MPSp was chosen to be used in the next phase of validation. The scale can be viewed in the Appendix section.

#### Study 2

##### Participants

In this study, the positively worded scale version demonstrating the best psychometric properties in Study 1 was tested on a sample of local government employees. The questionnaires were sent out to 2,650 recipients across the country. Four hundred and forty-one questionnaires were returned. Of them, nine were empty and 12 had so much missing data that they were excluded from statistical analysis. In the remaining sample (*n*_4_ = 420), the mean age for respondents was 45.74 years (*SD* = 10.84) and the proportion of women was 54.88 %. In n_4_, the educational level was represented as follows: PhD or higher = 0.03%, bachelor = 40.5%, master = 38.2%, and other = 21.3%. As the loading values in Study 1 were homogeneous, the loading average was above 0.6, and there are three variables per factor, the sample size is perfectly sufficient for a CFA. The minimally acceptable sample size in this scenario is n ≥ 400 (Gagne and Hancock, 2006).

##### Data Analysis

As the EFA revealed a strong tendency toward unidimensionality in MPSp, the CFA model was fitted to both the two- and the one-factor solution. Tests were run in FACTOR using both MLR (robust maximum likelihood) and robust RDWLS to test model fit (Mîndrila, 2010; Finney and DiStefano, 2013; Li, 2016a,b). Cutoff values were based on conservative recommendations (Schermelleh-Engel et al., 2003; Brown, 2014). Significant χ^2^ test results have been ignored in many psychometric studies (Heene et al., 2011). However, Ropovik (2015) rightly points out that the χ^2^ test is the only statistical test of model-data fit. Since in our study the sample size is not big, the model is simple, and the factor loadings are adequately high, the χ^2^ test results provide valuable information. χ^2^ test takes on even bigger significance if we consider that earlier studies found that fit indices such as TLI, CFI, or RMSEA might not be trusted with small sample sizes (Ainur et al., 2017). Ts-RMSEA and Ts-CFI are values gained through equivalence testing. This method typically avoids Type I and Type II errors that commonly occur during conventional hypothesis testing (Yuan et al., 2016). Normed/relative χ^2^ (χ^2^/df) values below 2 are accepted as a conservative cutoff (Bigras et al., 2017).

##### Results and Discussion

Table 5 shows the fit indices for the one- and two-factor model of MPSp.

**Table 5:** CFA fit indices.

**Fit indices**	**Cutoff values**	**One-factor solution**		**Two-factor solution**	
		**RML**	**RDWLS**	**MLR**	**RDWLS**
NT^a^ χ^2^	χ^2^/df <2	132.42	–	2.87	–
df		9	–	4	–
p		0.00	–	0.58	–
RMVA^b^ χ^2^	χ^2^/df <2	89.10	12.90	4.71	4.48
Df		9	9	4	4
P		0.00	0.00	0.32	0.34
MFF^c^ χ^2^	χ^2^/df <2	–	73.36	–	1.45
df		–	9	–	4
*p*		–	0.00	–	0.83
RMSEA^d^	≤ 0.06	0.14	0.17	0.021	0.01
Ts-RMSEA^e^	≥0.94	0.17	–	0.07	–
TLI^f^	≥0.95	0.80	0.77	0.99	0.99
CFI^g^	≥0.95	0.88	0.86	0.99	0.99
Ts-CFI^h^	≥0.94	0.79	–	0.98	–
RMSR^i^	≤0.05	0.11	0.12	0.01	0.01

a *NT, Normal Theory*;

b *RMVA, robust mean and variance-adjusted*;

c *MFF, minimum fit function*;

d *RMSEA, root mean square error of approximation*;

e *Ts-RMSEA, equivalence-tested RMSEA*;

f *TLI, Tucker–Lewis index*;

g *CFI, comparative fit index*;

h *Ts-CFI, equivalence-tested CFI*;

i *RMSR, root mean square of residuals*.

**Figure 3:**
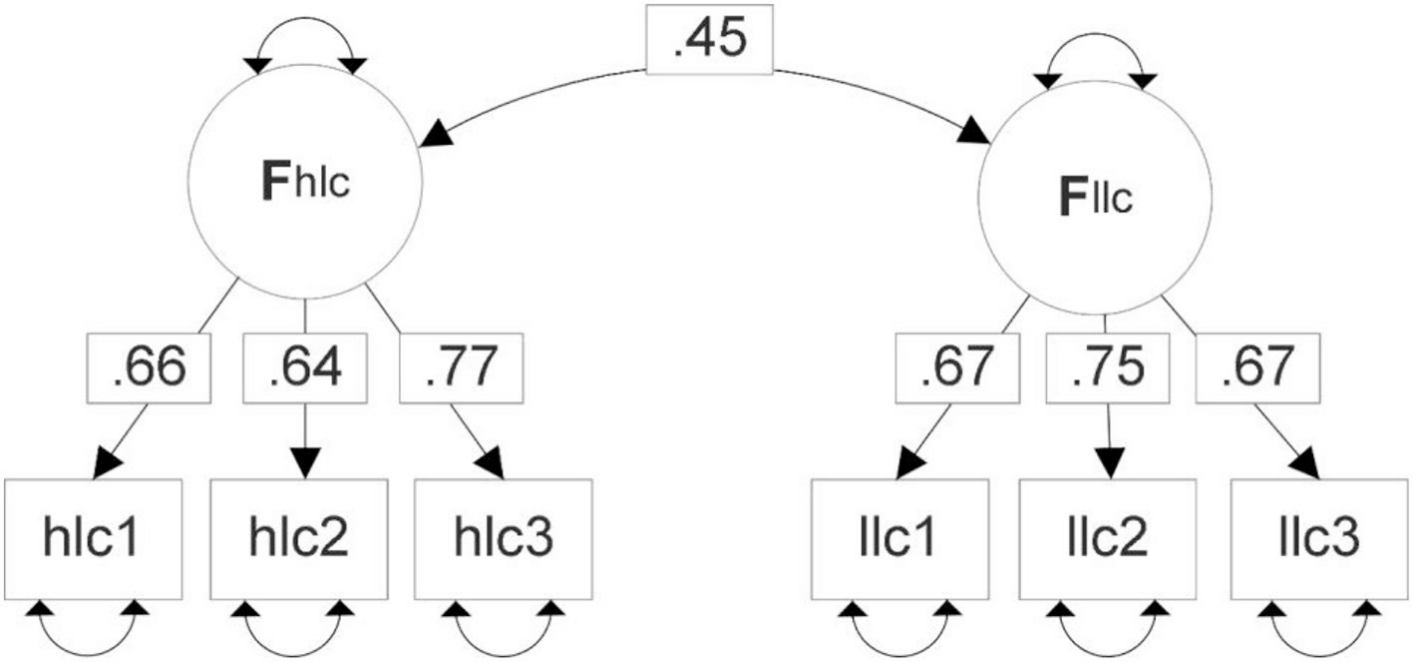
CFA model for MPSp.

None of the fit indices for the one-factor solution meet generally adopted cutoff criteria. Normed/relative χ^2^ (χ^2^/df) is not part of Table 5, but it is apparent that for the two-factor model all relative χ^2^ values fall below 2. It can be stated that the two-factor model of MPSp suggested by Study 1 has been validated by the CFA in Study 2. Figure 3 shows the CFA model for MPSp with factor loadings and interfactor correlation.

#### Study 3: Measurement Invariance Across Gender

##### Participants

In Study 3, students who have not taken part in Study 1 studying at the University of Debrecen were tested to examine measurement invariance across gender. The potential respondents received the link to the online questionnaire in an e-mail. Seven hundred and sixty-seven e-mails were sent out, and 271 students completed the questionnaire. Eight questionnaires were empty, six questionnaires did not contain information for gender, and seven questionnaires contained so much missing data that they were excluded from the analysis. In n_5_, the mean age for respondents was 20.92 years (*SD* = 4.74), and the proportion of women was 61.6%. There was no significant difference in demographics between those who did not send back the questionnaires and those who were included in the study. In n_5_, the three training programs were represented as follows: 2-year vocational = 31.2%, bachelor = 39.6%, and masters = 29.2%. AFIs (approximate fit indices) such as CFI are not very sensitive to sample size. χ^2^ tends to over-reject models as sample size increases; thus, *n*_5_ = 250 can be considered to be suitable for the measurement invariance analysis (Putnick and Bornstein, 2016).

##### Data Analysis

Data analysis was carried out using the Lavaan R package. The Lavaan package has been used extensively by numerous psychometric validation studies before (Liu et al., 2017; Sinval et al., 2018; Zhang et al., 2019). WLSMV and MLR estimators are suggested for measurement invariance tests on ordered polytomous data, with WLSMV being a better estimator of overall model fit and MLR a better estimator of change in fit (Sass et al., 2014; Li, 2016b; Koziol and Bovaird, 2018). The MPS_p_ scale and its two subscales F_hlc_ and F_llc_ were tested for invariance across gender. The four steps of testing measurement invariance with MLR, treating variables as continuous, are configural, weak, strong, and strict. With WLSMV, treating variables as ordered categorical, the four models are termed baseline, loading, threshold, and unique factor models, respectively (Liu et al., 2017). It has to be noted that in psychometric testing, strict/unique invariance is rarely reached or needed (Eriksson and Boman, 2018). Although values of RMSEA and SRMR were reported, CFI is considered to be the most reliable change-in-fit indicator (Cheung and Rensvold, 2002); thus besides the chi-square tests (Satorra and Bentler, 2001), it was used as the main determinant of invariance (Meade et al., 2008). Cutoff values suggested by Schermelleh-Engel et al. (2003) were used. The nested models were tested using the “measurementInvariance” function of the “semTools package” in R statistical software (Jorgensen et al., 2019).

##### Results and Discussion

Fit indices of the configural/baseline model were good. Evaluation was based on cutoff values (*CFI* ≥ 0.97, *TLI* ≥ 0.97, *RMSEA* ≤ 0.05, *SRMR* ≤ 0.05). The results with the MLR estimator were *CFI* = 1.00, *TLI* = 1.00, *RMSEA* = 0.00, and *SRMR* = 0.02 and with the WLSMV estimator *CFI* = 0.99, *TLI* = 0.99, *RMSEA* = 0.02, and *SRMR* = 0.03.

Table 6 shows the results of the scaled chi-square difference test. Evaluation was based on ΔCFI cutoff values (ΔCFI ≤ 0.01). For the nested models with the MLR estimator, CFI values ranged 0.99–1, ΔCFI values −0.002–0.000, with the WLSMV estimator CFI values ranged 0.97–0.99, ΔCFI values −0.01–−0.02. With the MLR estimator, RMSEA values ranged 0.000–0.026 and with the WLSMV estimator 0.023–0.055. SRMR values with the MLR estimator were 0.037–0.067, while with the WLSMV estimator, they ranged 0.032–0.057. According to the cited cutoff recommendations and ΔCFI values by the WLSMV estimator, strict/unique factor invariance does not hold. Based on values by the MLR estimator, however, strict/unique invariance seems to hold.

**Table 6:** Scaled chi-square difference test of MPS_p_ (method = “satorra.bentler.2001”).

**Models**	***χ***^****2****^		**df**		***p***		**Δ*****χ***^****2****^		**CFI**	
	**mlr**	**wlsmv**	**mlr**	**wlsmv**	**mlr**	**wlsmv**	**mlr**	**wlsmv**	**mlr**	**wlsmv**
Model 1	14.78	17.03	16	16	0.54	0.38	–	–	1	0.99
Model 2	19.98	21.41	20	20	0.45	0.37	−5.2	−4.38	1	0.99
Model 3	24.88	26.46	24	24	0.41	0.33	−4.90	−5.50	0.99	0.99
Model 4	27.81	35.68	26	26	0.36	0.09	−2.93	−9.22	0.99	0.97

*Model 1, configural/baseline; Model 2, weak/loading; Model 3, strong/threshold; Model 4, strict/unique factor*.

Table 7 shows the results of the scaled chi-square difference test for MPSp subscales. Evaluation was based on ΔCFI cutoff values (ΔCFI ≤ 0.01).

**Table 7:** Scaled chi-square difference test of F_hlc_ and F_llc_ subscales (method = “satorra.bentler.2001”).

**Models**	***χ***^****2****^		**df**		***p***		**Δ*****χ***^****2****^		**CFI**	
	**mlr**	**wlsmv**	**mlr**	**wlsmv**	**mlr**	**wlsmv**	**mlr**	**Wlsmv**	**mlr**	**Wlsmv**
**F**_**hlc**_ **subscale**										
Model 1	0.00	0.00	0	0	–	–	–	–	1.00	1.00
Model 2	1.72	1.53	2	2	0.42	0.46	−1.72	−1.53	1.00	1.00
Model 3	2.49	1.91	4	4	0.64	0.75	−0.77	−0.38	1.00	1.00
Model 4	4.76	8.87	5	5	0.44	0.11	−2.27	−6.96	1.00	0.96
**F**_**llc**_ **subscale**										
Model 1	0.00	0.00	0	0	–	–	–	–	1.00	1.00
Model 2	2.36	2.07	2	2	0.30	0.35	−2.36	−2.07	0.99	1.00
Model 3	6.20	6.76	4	4	0.18	0.14	−3.84	−4.69	0.97	0.98
Model 4	8.31	8.81	5	5	0.14	0.11	−2.11	−2.05	0.96	0.97

*Model 1, configural/baseline; Model 2, weak/loading; Model 3, strong/threshold; Model 4, strict/unique factor*.

Testing the F_hlc_ with the MLR estimator, CFI was 1 at all levels of the nested model, while with the WLSMV estimator, CFI values ranged 0.96–1.00, ΔCFI values ranged −0.035–0.000. With the MLR estimator, RMSEA values ranged 0.00–0.03, and with the WLSMV estimator, RMSEA values were 0.00 at all levels of the nested model. SRMR values with the WLSMV estimator ranged 0.00–0.05, and with the MLR estimator 0.00–0.06. Based on ΔCFI values by the WLSMV estimator, strict/unique factor invariance does not hold. Based on the values by the MLR estimator, however, strict/unique invariance seems to hold.

In the case of the F_llc_, the MLR estimator CFI values ranged 0.96–1.00 and with WLSMV 0.98–1.00. ΔCFI values for the MLR estimator were −0.003–−0.010 and for the WLSMV estimator −0.006–0.000. RMSEA values with the MLR estimator ranged 0.00–0.07, while with the WLSMV estimator 0.00–0.07. SRMR values with the MLR estimator ranged 0.00–0.06 and with the WLSMV estimator 0.00–0.05. Based on ΔCFI values by both WLSMV and MLR estimators, neither strong nor strict invariance holds.

The high-level construal subscale (F_hlc_) and the low-level construal subscale (F_llc_) did not demonstrate significant differences in preserving measurement invariance across gender.

#### Study 4: Concurrent Validity

##### Participants

In Study 4, MPSp was included in the validation questionnaire with 10 concurrent instruments. This validation questionnaire was sent out to 624 principals and teachers of secondary schools. One hundred and sixty-one questionnaires were returned in the 2 weeks the survey was open. Fifteen questionnaires contained no information at all, 12 were excluded because the respondents used the same scale points throughout the test, and six had no demographic information and a lot of missing answers. The statistical analysis was carried out on the remaining sample (*n*_6_ = 128). In n_v_, the mean age for respondents was 48.64 years (*SD* = 13.18), and the proportion of women was 89.80%. In n_6_, the educational level was represented as follows: PhD or higher = 1.2%, bachelor = 2.5%, master = 94.1%, and other = 2.2%. As the sample is relatively small, the 80% statistical power (two-tailed, α ≤ 0.05) of the Spearman's correlation is valid for correlation values *r*_*s*_ ≥ 0.25 (Bonett and Wright, 2000).

##### Data Analysis

Internal consistency and reliability of the concurrent instruments were checked to obtain a comparison with the findings of the original validation studies. For scales demonstrating a unidimensional character, composite reliability was computed. For scales not showing unidimensionality, subscale reliabilities are presented. The “laavan,” “userfreindlyscience,” and “effsize” R packages, FACTOR and SPSS version 25, were used to carry out the statistical analyses. As all instruments contain ordered categorical variables, scale scores were compared using Spearman's correlations (Yu et al., 2018). Correlations were interpreted using Cohen's (1992) recommendations (small = 0.10, medium = 0.30, and large = 0.50). Results about the relevant facets of mindfulness in earlier concurrent validity tests were considered to evaluate the validity of MPSp (Baer et al., 2004, 2008; Lilja et al., 2011; Christopher et al., 2012; Desrosiers et al., 2013; Tejedor et al., 2014; Aguado et al., 2015; Medvedev et al., 2018; Schutte and Malouff, 2018; Carpenter et al., 2019; Mattes, 2019; Sadowski et al., 2020).

*Instruments used in the study:* A six-item Nature Relatedness Scale (NR-6) is a brief measure of nature relatedness developed by Nisbet and Zelenski (2013). In our study, its reliability was good (ordinal Cronbach's alpha = 0.89), and the unidimensionality was confirmed (*UniCo* = 0.98, *ECV* = 0.87, *MIREAL* = 0.25).

*Questionnaire for Eudaimonic Well-Being (QEWB)* is a 21-item scale developed by Waterman et al. (2010) to measure well-being as interpreted by eudemonist philosophy, and the scale was found to be unidimensional. As in the original validation, the instrument showed an excellent reliability (ordinal Cronbach's alpha = 0.95), but did not prove to be unidimensional (*UniCo* = 0.88, *ECV* = 0.76, *MIREAL* = 0.27).

*MAAS 10-item shortened scale* was created by Chiesi et al. (2017) by omitting badly performing items of the original MAAS. The IRT (Item Response Theory) analysis revealed that the ten-item version had good psychometric properties. In our study, its reliability was good (ordinal Cronbach's alpha = 0.89), and the unidimensionality was confirmed (*UniCo* = 0.97, *ECV* = 0.88, *MIREAL* = 0.21).

*Freiburg Mindfulness Inventory (FMI) 14-item shortened form* is a scale developed by Walach et al. (2006) from the 30-item Freiburg Mindfulness Inventory (FMI). It showed good psychometric properties and was tentatively termed as unidimensional. In our study, the scale reliability was good (ordinal Cronbach's alpha = 0.87) and unidimensionality was confirmed (*UniCo* = 0.94, *ECV* = 0.80, *MIREAL* = 0.28).

*CAMS-R 10-item shortened version* was developed by Feldman et al. (2007). In the validation, one higher-order factor and four first-order factors were revealed. In our study, it showed good reliability (ordinal Cronbach's alpha = 0.86) and its deeper level unidimensional character was also confirmed (*UniCo* = 0.93, *ECV* = 0.81, *MIREAL* = 0.27).

*FFMQ 15-item shortened version* is a short version of the 36-item FFMQ developed by Baer et al. (2008). In our study, no unidimensional character was demonstrated (*UniCo* = 0.67, *ECV* = 0.60, *MIREAL* = 0.41). Subscale reliabilities as measured by ordinal Cronbach's alpha were acceptable (*Observe* = 0.72; *Act.aware* = 0.81; *Non-react* = 0.64; *Non-judge* = 0.75; *Describe* = 0.81).

*PHLMS* is a 20-item bidimensional scale created by Cardaciotto et al. (2008). In the present study, the reliability of both the Awareness (ordinal Cronbach's alpha = 0.87) and the Acceptance (ordinal Cronbach's alpha = 0.88) subscales was good. The instrument was not found to be unidimensional (*UniCo* = 0.74, *ECV* = 0.55, *MIREAL* = 0.46).

*Social Connectedness Scale (SCS)* is one of the two subscales of the Social Belongingness Scale developed by Lee and Robbins (1995). This eight-item measure demonstrated an excellent reliability (ordinal Cronbach's alpha = 0.95), and unidimensionality was confirmed (*UniCo* = 0.99, *ECV* = 0.95, *MIREAL* = 0.18).

*Resistance to Change Scale* (RCS) is an 18-item instrument created by Oreg (2003). In our study, the scale reliability was excellent (ordinal Cronbach's alpha = 0.92), and unidimensionality was confirmed (*UniCo* = 0.92, *ECV* = 0.87, *MIREAL* = 0.24).

*Hospital Anxiety and Depression (HAD) Scale* is a 14-item instrument that was originally developed for clinical populations (Zigmond and Snaith, 1983), but later tested successfully on general populations as well (Mykletun et al., 2001; Djukanovic et al., 2017). In our study, it showed an excellent reliability (ordinal Cronbach's alpha = 0.91) and was found to be unidimensional (*UniCo* = 0.94, *ECV* = 0.84, *MIREAL* = 0.25).

The NR-6, the EWBQ, and the RCS were included in the concurrent study with further research objectives in mind. With the escalating global socioecological crisis, certain skills and values such as the attitude to nature, eudemonic values, or the ability to change will become crucially important. In recent years, mindfulness as a possible catalyst of the transition to a sustainable lifestyle has been receiving increasing attention from researchers.

##### Results and Discussion

Table 8 contains concurrent correlations between MPSp and the seven concurrent instruments.

**Table 8:** Concurrent correlations.

**Scales and subscales**	**F_**hlc**_**	**F_**llc**_**	**MPS_**p**_**
NR-6	0.19^**^	0.45^**^	0.38^**^
QEWB	0.27^**^	0.30^**^	0.36^**^
MAAS	0.25^**^	0.22^**^	0.27^**^
FMI	0.32^**^	0.37^**^	0.42^**^
CAMS-R	0.31^**^	0.32^**^	0.37^**^
CAMS-R at.^a^	0.24^**^	0.26^**^	0.28^**^
CAMS-R pr.f.^b^	0.28^**^	0.26^**^	0.30^**^
CAMS-R aw.^c^	0.22^**^	0.27^**^	0.29^***^
CAMS-R ac.^d^	0.20^**^	0.22^**^	0.25^**^
FFMQ	0.30^**^	0.45^***^	0.45^**^
FFMQ obs.^e^	0.37^**^	0.68^**^	0.61^**^
FFMQ n.j.^f^	0.05	0.00	0.00
FFMQ n.r.^g^	0.18^*^	0.21^*^	0.23^**^
FFMQ a.a.^h^	0.24^**^	0.28^**^	0.3^***^
FFMQ des.^i^	0.03	0.06	0.07
PHLMS	0.26^**^	0.32^**^	0.35^**^
PHLMS awa.^j^	0.34^**^	0.59^**^	0.53^**^
PHLMS acc.^k^	0.00	−0.12	−0.05
SCS	−0.12	0.00	−0.09
RCS	−0.01	−0.15	−0.11
RCSrout.seek^l^	0.09	−0.15	−0.04
RCSemot.react.^m^	−0.05	−0.04	−0.07
RCSshort.t.foc.^n^	**−0.13**	−0.24^**^	−0.23^**^
RCScogn.rig.^o^	0.00	−0.09	−0.05
HAD	**−0.12**	−0.22^*^	−0.21^*^
HAD anx.^p^	**0.09**	−0.19^*^	−0.17
HAD dep.^q^	**−0.14**	−0.22^*^	−0.22^*^

* *p ≤ 0.05 level of significance*;

** *p ≤ 0.01 level of significance*;

*** *p ≤ 0.001 level of significance*;

a *at., attention*;

b *pr.f., present focus*;

c *aw., awareness*;

d *ac., acceptance*;

e *obs., observing*;

f *n.j., non-judging*;

g *n.r., non-reacting*;

h *a.a., acting with awareness*;

i *des., describing*;

j *awa., awareness*;

k *acc., acceptance*;

l *rout.seek, routine seeking*;

m *emot.react., emotional reaction*;

n *short.t.foc., short-term focus*;

o *cogn.rig., cognitive rigidity*;

p *anx., anxiety*;

q *dep., depression. Bold values are the ones where the low level construal variables (factor) demonstrated a significantly bigger correlation with the concurrent instruments than the high level construal factor*.

In the case of QEWB, FMI, SCS, and RCS, no subscale scores were calculated as they were found to be unidimensional both in the original study and in ours. Correlations range from small to large with most values being in the small and medium range. The F_llc_ of MPSp had a medium correlation with the composite scores of FFMQ, and large correlations with the Observing facet of FFMQ and the Awareness subscale of PHLMS. The strongest correlations of the F_hlc_ subscale of MPSp were in the medium range with FMI and FFMQ composite scores and the Observing facet of FFMQ. MPSp subscale and composite scores had a small correlation with MAAS. Acting with awareness is moderately correlated with MPSp and both of its subscales. Interestingly, the F_llc_ of MPSp, consisting of items closely corresponding to FFMQ Observing variables, is weakly but significantly negatively correlated with both anxiety and depression variables of HADS. The Non-judging FFMQ facet is uncorrelated with F_hlc_ consisting of Acting with awareness type of variables, while in previous studies, they are typically positively correlated. The Describe facet of FFMQ in our study is uncorrelated with either subscales of MPSp.

The positive correlation between MPS_p_ composite scores and NR-6 supports the earlier literature; however, the correlation is higher than what is reported in earlier studies (*r* between 0.17 and 0.25). The strong correlation of the F_llc_ subscale with NR-6 seems to be in line with recent findings of similar studies where Observing-type items of existing scales were used.

There is no earlier data in the literature about the relationship between social connectedness and Observing-type (low-level construal) and Acting with awareness-type (high-level construal) mindfulness scale items. In our study, except for the weak correlation of the short-term focus subscale of RCS with the F_llc_ subscale, these constructs were found to be uncorrelated with MPS_p_ subscales.

### General Discussion

The primary goal of this study was to carry out the initial validation of a six-item trait mindfulness measure consisting of three high-level and three low-level construal scale items. While the five-item MAAS and six-item MAAS are valid short instruments to measure a dimension of trait mindfulness, all of their items are general (high-level construal) statements about certain MP skills in general. As a unique contribution to existing mindfulness measurement methodology, our research used an equal number of high-level construal (not action-specific) and low-level construal (action-specific) items to test whether the construal level of scale items significantly affects respondent's recollection of mindful moments and thus the psychometric properties of the scale.

The MPS_p_ demonstrated good psychometric properties with above 0.6 homogeneously distributed factor loadings across the two construal-level subscales. As a contribution to existing statistical methodology in mindfulness research, our study used various EFA indices (Ferrando and Lorenzo-Seva, 2018) relatively new to mindfulness literature. The stable factor structure found in Study 1 was confirmed in the CFA tests in Study 2 and the measurement invariance tests of Study 3. Although, based on the EFA indices, the high-level construal subscale performed better in Study 1, this difference was not consistent across the other studies and samples; thus, statistical point of view nothing conclusive can be stated about how the two different construal-level subscales behave. Including concrete, action-specific items in the scale proved to be an incremental value over the short MAAS scale versions as there is a marked difference in how high- and low-level construal items relate to certain concurrent instruments. In Study 4, correlations with well-known mindfulness instruments are of the magnitude and in the direction that had been expected. The strong correlation of the low-level construal subscale of MPS_p_ (F_llc_) with the Observing subscale of FFMQ and the Awareness subscale of PHLMS can be attributed to the fact that items of these subscales are very similar.

Concurrent correlations showed a consistent pattern with the low-level construal subscale (F_llc_) being the stronger predictor of the concurrent instruments except for the present focus subscale of CAMS-R where the high-level subscale (F_hlc_) had a slightly stronger correlation. The difference between the two subscales of MPS_p_ was especially marked when correlated with NR-6, the Observing subscale of FFMQ, the Awareness subscale of PHLMS, the Short term focus subscale of RCS and the HADS and its two subscales Anxiety and Depression.

The relatively strong correlation between F_llc_ and NR-6 can only be hypothetically explained as there are no prior studies on the relationship between nature-relatedness and the construal level of mindfulness scale items. A possible explanation can be the fact that nature is something to be enjoyed physically in its particularity and concreteness, that is, with a low-level construal mindset. The everyday activities described in the F_llc_ scale items prime the respondents to adopt a low-level construal type of thinking. In their study introducing the NR-6 scale, Nisbet and Zelenski (2013) argue that a higher level of nature relatedness corresponds to more sustainable behavior. From this, it might logically follow that F_llc_ could be a good predictor of sustainable behavior. However, in an earlier study, Amel et al. (2009) found that while the Acting with awareness facet of FFMQ positively correlated with sustainable behavior, Observing items were uncorrelated. Further research is definitely warranted into how F_hlc_ and F_llc_ items are related to sustainable behavior as there are several studies emphasizing the role mindfulness could play in transforming our unsustainable lifestyle (Wamsler and Brink, 2018; Thiermann and Sheate, 2020).

While there are several conceptual articles arguing for mindfulness being a catalyst of change (Gärtner, 2013), original research articles on the topic are virtually non-existent. As a result of this, our findings can only be explained tentatively. The fact that the low-level construal subscale (F_llc_) negatively correlates with the Short-term focus subscale of RCS might be attributable to the fact that people with more abstract thinking are more likely to see the “wider picture,” which means that they might not see changes as something inherently bad. Further research is needed to verify this hypothetical proposition.

As for the relationship between mindfulness in general and certain facets of it and various eudemonic well-being measures, the research results in the literature so far are controversial. Baer et al. (2004) found that the Observe facet of FFMQ did not have a significant relationship with eudemonic well-being and Caldwell et al., 2010) reported a positive correlation only in meditating samples. In Hollis-Walker and Colosimo's (2011) study, only the Acting with awareness and Describing facets of FFMQ had a significant correlation with psychological well-being. Baer et al. (2008) reported that with the exception of the Observing facet, all other facets of FFMQ were related to psychological well-being. Certainly, these comparisons have to be taken with caution as the PWB scales used in those studies are not identical with the QEWB used in our study.

As there are no prior data on how the SCS relates to facets of mindfulness, only indirect comparisons are possible with studies using similar instruments. In Adair, Fredrickson, Castro-Schilo, Kim and Sidberry (2018) study, composite FFMQ mindfulness scores were positively correlated with pro-social behavior. It has to be noted that the FFMQ version they used was without the Observing subscale. In our study, the Describing, Acting with awareness, and Non-judging facets of FFMQ were moderately correlated (*r* = 0.25–0.39, *p* ≤ 0.05) with SCS composite scores. Both MPS_p_ subscales are uncorrelated with SCS. In the previous chapter, it was pointed out that the Observing facet of FFMQ, being similar to the F_llc_ subscale, also has an ambiguous relationship with anxiety and depression measures.

In summary, it can be stated that this study has not found evidence that the construal level of trait mindfulness scale items has a significant effect on the recollection of mindful episodes by respondents. Both the high-level construal (F_hlc_) and the low-level construal (F_llc_) subscales demonstrated similar factorial stability, reliability, and invariance. However, there were remarkable differences between them in the concurrent validity test. Consequently, our hypothesis that the high- and low-level construal items would behave differently in the statistical analysis has been only partly confirmed. Our hypothesis, predicting that in spite of the single facet construct of MP and the expected unidimensional tendencies, items would gravitate toward two hidden variables according to their level of construal, has been confirmed.

## Conclusions

Using samples from various base populations, ample evidence was found that the positively worded scale version MPSp is a valid instrument with clear and stable factor structure across samples and gender, good reliability, and adequate concurrent validity. The bifactorial structure was confirmed in two different samples through EFA and CFA. In spite of the fact that a bifactorial structure was consistently evidenced in Study 1, Study 2, and Study 3, indices (UniCo, ECV, MIREAL) used to measure the construct's closeness to unidimensionality and the interfactor correlation signaled an underlying common factor. This result lends support to our initial claim hypothesizing that both high- and low-level construal scale items in MPSp tap into a central aspect of the complex construct termed “Mindful Presence,” which encompasses skills and abilities to bring attention to and anchor it in the present moment. We feel that our study adds to the existing literature in several ways. Besides introducing a really short but valid trait mindfulness measure, we directed attention to the yet unresearched issue of the construal level of scale items. The MPSp scale's main fields of application can be validation studies where several concurrent instruments are used at the same time as well as for target populations with very limited time for self-report tests.

### Limitations and Further Research

In Study 1, the three samples were drawn from the same base population of students of economics at the University of Debrecen with no significant difference between the samples regarding age and gender makeup; however, group effects cannot be ruled out with certainty. While in Study 1 and Study 3, the age and gender of students who did not send back the questionnaire were known, in Study 2 and Study 4, it could not be determined, which raises the possibility of these samples being selectively biased. Although diverse samples were used, in the future the scale should be tested on general and clinical populations to see if results can be replicated. The sample in Study 4 (n_6_) was relatively small, reducing the statistical power of the Spearman correlations under a given threshold of *r*_s_ value. Ten concurrent instruments were incorporated into this initial validation process, and although short-scale versions were used, respondent fatigue may have had an impact on scores. The response rate was relatively low in all four studies. One possible explanation is the lack of financial or other rewards often granted to student respondents. In the case of the two adult samples, limited free (Study 2 and Study 4) time and the length of the questionnaire (Study 4) might have been a possible cause of the low response rate. This validation study is based on Classic Test Theory (CTT). IRT in future research can provide important information (i.e., item difficulty) not addressed by CTT (Sébille et al., 2010; Ye et al., 2018, 2019). Even though MPSp items have been categorized into construal-level groups based on whether they are action-specific or general statements about mindfulness, as construal level is a continuum, the procedure can be considered arbitrary.

In the future, MPSp's relationship with other instruments could also be tested. Because of the dynamism of mindful and mindless moments in non-meditating samples and the fact that self-report instruments can only measure respondents' recollection of mindful experience at a particular moment, trait mindfulness can only be established with certainty based on time-series data of repeated measures done on the same sample; thus, the notion “trait” should be taken with caution. The application of generalizability theory (Truong et al., 2020; Ye et al., 2020) might help to grasp the trait or state nature of MPSp scale items in future studies.

## Data Availability Statement

The original contributions presented in the study are included in the article/Supplementary Material, further inquiries can be directed to the corresponding author/s.

## Ethics Statement

The studies involving human participants were reviewed and approved by University of Debrecen Clinical Center, Regional and Institutional Committee of Scientific Ethics. The patients/participants provided their written informed consent to participate in this study.

## Author Contributions

AL, DK, RO, SS, ÉB, ZB, and AM: conceptualization, investigation, and writing—review and editing. AL, DK, RO, and SS: formal analysis, methodology, and writing—original draft. ÉB and ZB: funding acquisition. ÉB, ZB, and AM: project administration, resources, and visualization. AL, DK, RO, SS, and AM: validation. All authors commented on previous versions of the manuscript, read, and approved the final manuscript.

## Conflict of Interest

The authors declare that the research was conducted in the absence of any commercial or financial relationships that could be construed as a potential conflict of interest.

## Appendix

Below is the English translation of MPSp.

### MPSp

Imagine that you are **at home, alone, spending your free time**. How much are the following statements true for you? Check one of the six options.

The six-scale points were the following: almost always, very frequently, somewhat frequently, somewhat infrequently, very infrequently, and almost never.

- When I'm doing routine activities, I focus only on what I am doing.
- When I'm doing everyday tasks, I re-live the experience with full attention and awareness every time.
- I don't rush routine activities only to be able to do something more interesting afterward.
- When I'm having a shower or a bath, I pay all my attention to that activity. For example, the sound of the water, the feeling as it runs down my body, the way my body reacts etc.
- When I'm eating, I pay all my attention to eating. For example, how the flavors of the food appear while chewing, how the texture of the food changes, the feeling when I swallow it, etc.
- When I'm getting dressed, I focus all my attention on that activity. For example, the smell of the clothes, the material touching my skin, the touch of the buttons etc.
